# Development and validation of a prediction model for urinary tract infection in older patients with type 2 diabetes mellitus

**DOI:** 10.3389/fendo.2026.1706338

**Published:** 2026-01-27

**Authors:** Lin Yang, Jian Yin, Zhengxiong Yang, Jing Liu

**Affiliations:** 1 Hospital-Acquired Infection Control Department, The Affiliated Lianyungang Hospital of Xuzhou Medical University, Lianyungang, China; 2 Department of Endocrinology, The Affiliated Lianyungang Hospital of Xuzhou Medical University, Lianyungang, China

**Keywords:** nomogram, older, prediction model, risk factors, type 2 diabetes mellitus, urinary tract infection

## Abstract

**Objective:**

To analyze the pathogen distribution of older type 2 diabetes mellitus (T2DM) patients with urinary tract infection (UTI) and to develop and validate the feasibility of a nomogram risk prediction model for older T2DM patients with UTI.

**Methods:**

We retrospectively analyzed clinical data from older patients with T2DM admitted to the Department of Endocrinology of The First People’s Hospital of Lianyungang City from December 2023 to December 2024. Random number sequences were generated using R software, and all patients were assigned to the modeling cohort and validation cohorts in a ratio of 7:3 through simple random sampling. We compared baseline features between training and validation sets using appropriate statistical tests. We employed univariate and multivariate logistic regression models to identify factors independently associated with UTI in older patients with T2DM. Subsequently, we developed a nomogram prediction model, which was then validated using the validation group. Four methods of statistics, namely, the Hosmer–Lemeshow goodness-of-fit test (H-L test), subjects’ work characteristic curve and area under the curve (AUC), calibration curve, and clinical decision curve (DCA), were comprehensively applied to evaluate the model’s fit, discrimination, calibration, and clinical utility.

**Results:**

Among 521 older patients with T2DM, 82 developed UTI, with an incidence of 15.74%. Logistic regression analyses identified female (odds ratio [OR] = 2.53, 95% confidence interval [CI]: 1.25–5.12, *P* = 0.011), HbA1c (OR = 1.35, 95% CI: 1.15–1.59, *P* = 0.001), SGLT-2i in previous year (OR = 2.29, 95% CI: 1.19–4.41, *P* = 0.013), indwelling urinary catheter (OR = 3.04, 95% CI: 1.02–9.23, *P* = 0.048), and UTI in previous year (OR = 5.22, 95% CI: 2.22–12.25, *P* = 0.001) as independent risk factors for older T2DM patients with UTI. The AUC was 0.779 (95% CI: 0.703–0.855) in the training group and 0.793 (95% CI:0.691–0.896) in the validation group. The H-L test results for the training (χ² = 9.834, *P* = 0.277) and validation (χ² = 5.432, *P* = 0.711) groups indicated good goodness-of-fit for the prediction model, with the internal validation curve demonstrating satisfactory performance.

**Conclusion:**

Establishing this simple, intuitive nomogram prediction model enables the early identification of risk factors in older T2DM patients with UTI. Based on readily accessible clinical variables, this model facilitates early risk stratification and promotes preventive interventions in a timely manner, ultimately reducing UTI incidence in clinical practice. Furthermore, the identified pathogen distribution and antimicrobial susceptibility profiles directly support targeted UTI treatment in this high-risk population, further enhancing the clinical utility of the predictive model and potentially improving patient outcomes.

## 1 Introduction

Type 2 diabetes mellitus (T2DM) is a prevalent chronic metabolic disease among older adults. With the increase in aging population, the number of older people with T2DM has more than increased fourfold in the past 30 years (1). Older patients with T2DM often exhibit reduced immune function, and chronic hyperglycemia further impairs immune cell activity (2–4), predisposing them to the risk of various types of infections, of which urinary tract infection (UTI) is the most common (5). Studies have shown that the incidence of UTI in older patients with T2DM ranges from 11.5 to 43% (6, 7). T2DM is associated with a 1.5–4-fold increased risk of UTI relative to nondiabetic controls (8, 9). UTI increases the difficulty of glycemic control in older patients with T2DM, affects their quality of life, and predisposes them to risk of secondary infections such as pyelonephritis and sepsis, which further increases the risk of death (10–12). Early risk prediction and timely intervention of older T2DM patients with UTI are of immense significance in preventing their occurrence.

Risk prediction modeling refers to the combination of disease-related predictors, the use of statistical methods to calculate the weight of each predictor, and the establishment of a model to predict the risk of disease occurrence (13). Although existing models possess certain clinical value, they still exhibit notable limitations in predictive performance, methodological rigor, generalization capability, and practicality. For instance, the questionnaire-based model developed by Venmans et al. (14, 15) demonstrated moderate discriminatory ability [area under the curve (AUC): 0.72 and 0.79] and reported Hosmer–Lemeshow (H-L) test results; however, its evaluation methods remain relatively constrained, lacking comprehensive assessments of clinical utility or internal validation. Although the model proposed by Tu Jing et al. (16) reported a high AUC value (0.978), it was constrained by a small sample size (N = 182), which was below the minimum sample size required for prediction model establishment. This study also failed to report assessments of model calibration, clinical utility, or internal validation procedures (16). Furthermore, although the machine learning model developed by Yu Xiong et al. (17) achieved high accuracy (AUC: 0.978), it requires the input of 106 initial variables and performing complex computational interpretations. This poses notable challenges for integrating the model into routine clinical workflows, particularly in resource-constrained settings. Furthermore, these models are primarily derived from Western primary care populations (14, 15) or tertiary care hospitals in major Chinese cities (16, 17), raising questions about their applicability across different tiers and resource levels of Chinese healthcare institutions.

This study aims to investigate the current status of older T2DM patients with UTI, analyze their risk factors, and establish a concise and intuitive predictive map based on readily available clinical variables to assess UTI risk in this high-risk population. The model is designed to guide early identification and intervention for high-risk groups across different hospital settings. We will also elucidate the pathogen spectrum and antimicrobial susceptibility features of UTI in this population, providing a pathological basis for clinical prevention and reference for initial empirical treatment.

## 2 Materials and methods

### 2.1 Study design and participants

We conducted a single-center retrospective study at The First People’s Hospital of Lianyungang, enrolling consecutive older patients with T2DM who were hospitalized from December 2023 to December 2024. Patients were randomly assigned to a modeling cohort (N = 364, 70%) and a validation cohort (N = 157, 30%) in a 7:3 ratio using the simple randomization technique. The adequacy of randomization was assessed by comparing baseline features between the two cohorts. Each cohort was further stratified into UTI and non-UTI groups.

The inclusion criteria were as follows: (1) Age ≥ 60 years; (2) Meeting the diagnostic criteria for T2DM proposed by the Chinese Diabetes Prevention and Control Guidelines (2024 edition) (18): random fasting blood glucose (FBG) ≥ 11.1 mmol/L (measured at any time postprandially), or fasting FBG ≥ 7.0 mmol/L (after no caloric intake for at least 8 h), or 2-h postprandial blood glucose (2h-PBG) after an oral glucose tolerance test (OGTT) ≥ 11.1 mmol/L; (3) UTI was diagnosed based on established clinical and microbiological criteria (19). The clinical presentation of UTI involves manifestations of urinary tract irritation, substantiated by laboratory evidence. Microbiological confirmation requires a positive urine culture meeting the threshold for considerable bacteriuria, including asymptomatic bacteriuria as defined by guidelines.

The exclusion criteria were as follows: (1) Immune and coagulation dysfunction; (2) Malignant tumors; (3) Dysfunction of the heart, liver, kidney and other important organs; (4) Urinary tract deformities; (5) Hormonal or immunosuppressant therapy; (6) Infections in other parts of the body diagnosed at the time of hospital admission; (7) A documented history of urological surgical procedures; (8) Incomplete clinical records.

### 2.2 Data collection

#### 2.2.1 Pathogenic bacteria and drug resistance monitoring

Older T2DM patients with UTI were retained for pathogenic bacterial culture in the mid-morning urine specimens, which were placed in sterile tubes and then transported in a timely manner, and Gram-negative and Gram-positive bacilli were cultured using the MacConkey agar and blood agar mediums, respectively, whereas fungal cultures were performed using Sabouraud’s agar medium. Relevant quality-control tests such as aseptic tests and supportive growth tests were performed before experiments. Referring to CLSI M100-Ed33 guideline (20), the minimum inhibitory concentration of the target antimicrobial drug by pathogenic bacteria was determined by using the micro broth dilution method, and the drug sensitivity type was judged according to the fold point.

#### 2.2.2 Clinical data collection

Building upon a prior meta-analysis of risk factors for UTI among older patients with T2DM (21, 22), this study performed an expert consultation involving 16 multidisciplinary specialists from endocrinology, nephrology, urology, and hospital infection control departments with more than 15 years of relevant clinical experience. The consultation was administered via an email survey with a 100% response rate. Its primary objective was to identify potential, unreported risk factors by collecting expert opinions. The experts first reviewed the key findings of the prior systematic review and were then asked to independently propose additional risk factors potentially overlooked by the existing literature. These expert-suggested factors, combined with those identified in the meta-analysis, collectively formed a comprehensive set of candidate predictor variables for subsequent data collection and statistical model development. All clinical data of the patients were collected according to the Apricot Grove system of the hospital’s infection management department and the hospital’s electronic medical record system. Clinical data were collected by the relevant literature (21, 22) and also the results of the consultation, including general information, such as age, sex, duration of DM, body mass index (BMI); laboratory indicators, such as fasting plasma glucose (FBG), postprandial plasma glucose (2h-PG), glycosylated hemoglobin (HbA_1_c), blood urea nitrogen (BUN), serum creatinine (Scr), serum albumin, urinary protein, urinary microalbumin; treatment-related information, such as days of hospitalization, indwelling urinary catheter, and other relevant information.

### 2.3 Sample size justification

The sample size was determined based on the requirements for multivariate logistic regression and nomogram development, in accordance with the formula proposed by Peduzzi et al. (23) for sample size calculation with a binary outcome variable. Therefore, the minimum sample size for logistic regression was calculated using the following formula:


$$\text{N}=\frac{10\times \text{K}}{\text{P}}$$

Where N, K, and P represent the required sample size, number of variables, and proportion of positive results, respectively.

Assuming that five variables are ultimately included in the model and the UTI prevalence among older patients with T2DM in this study is 15.74%, it is indicated that the minimum sample size required for the training set was 318 patients. According to the requirements of logistic regression modeling, the training set accounts for approximately 7/10 of the total sample size, with the total sample size of patients being at least 455. Our final sample size of 521 patients exceeded this minimum requirement.

### 2.4 Variable assignment

The presence of UTI among older patients with T2DM was the dependent variable, and the rest of the indicators were independent variables, including sex, SGLT-2i in previous year (defined as having a documented prescription or use record of SGLT-2i at any time within the 12 months preceding admission, including current and past drug exposure), urinary protein, indwelling urinary catheter, UTI in previous year, and combined urinary stones. The remaining variables were treated as continuous variables. Assignment rules for categorical variables are shown in **Table 1**.

**Table 1:** Assignment rules for categorical data.

Indicator	Assignment
UTI	No = 0, Yes = 1
Sex	Male = 0, Female = 1
SGLT-2i in previous year	No = 0, Yes = 1
Urine protein	Negative = 0, Positive = 1
Indwelling urinary catheter	No = 0, Yes = 1
UTI in previous year	No = 0, Yes = 1
Combined urinary stones	No = 0, Yes = 1

### 2.5 Statistical analysis

Statistical description and data analysis were performed using SPSS 22.0, and model construction and validation were performed using R4.1.3. The Kolmogorov–Smirnov test was used to test the normality of the quantitative data distribution, and normally distributed data were expressed as the mean ± standard deviation ( $\bar{\text{x}}$ ± S) and compared between two groups using the independent-sample t-test. Quantitative data that were not normally distributed were presented as the median value and its interquartile range (*M* (*P*_25_, *P*_75_)) and compared between two groups using the Mann–Whitney U test. Categorical data were expressed as frequencies and percentages (%), and they were compared between groups using the χ^2^ test or Fisher’s exact test. A multicollinearity analysis was performed to eliminate interactions between factors before constructing regression models. Variables with *P* < 0.05 in the univariate analysis were included in the multivariate logistic regression analysis. Variables that remained statistically significant (*P* < 0.05) in the multivariate model were identified as independent predictors and used to develop the predictive model. The model’s performance was evaluated by plotting the Receiver Operating Characteristic (ROC) curve, AUC, and the H-L test. Internal validation was performed using the Bootstrap method (B = 1000), and calibration curves were plotted and decision curve analysis (DCA) performed to assess the model’s discriminatory power, goodness-of-fit, calibration, and clinical decision curve.

## 3 Results

### 3.1 Incidence of UTI and distribution of pathogenic bacteria in older T2DM patients

This study found that the UTI incidence among older patients with T2DM was 15.74% (82/521 patients). Among these patients with UTI, 89 pathogenic bacterial strains were identified. *Escherichia coli* was the most common pathogen, accounting for 49.44% of cases (**Table 2**).

**Table 2:** Distribution of pathogenic bacteria.

Pathogenic bacteria species	Number of strains (n = 89)	Percentage (%)
**Gram-negative bacteria**	**70**	**78.62**
*Escherichia coli*	44	49.44
*Klebsiella pneumoniae*	15	16.82
*Proteus mirabilis*	5	5.62
*Pseudomonas aeruginosa*	2	2.26
*Enterobacter cloacae*	1	1.12
*Stenotrophomonas maltophilia*	1	1.12
*Raoultella ornithinolytica*	1	1.12
*Proteus vulgaris*	1	1.12
**Gram-positive bacteria**	**13**	**14.60**
*Staphylococcus aureus*	5	5.62
*Streptococcus agalactiae*	5	5.62
*Bacillus subtilis*	1	1.12
*Enterococcus faecalis*	1	1.12
*Group A Streptococcus*	1	1.12
**Fungi**	**6**	**6.78**
*Candida glabrata*	4	4.52
*Candida albicans*	2	2.26

The bold entries (Gram-negative bacteria, Gram-positive bacteria, and fungi) represent the primary taxonomic categories of isolated pathogens, along with their respective isolation counts and proportions.

### 3.2 Drug sensitivity test of *Escherichia coli* in older T2DM patients with UTI

The drug sensitivity test results revealed that *Escherichia coli* was highly resistant to piperacillin and cefazolin, while exhibiting high susceptibility to ertapenem, imipenem, and meropenem (**Table 3**). These findings provide a reference for empirical antimicrobial selection in high-risk patients identified by the prediction model, especially when urine culture results are not immediately available.

**Table 3:** Drug susceptibility testing of *Escherichia coli* in UTI among older patients with T2DM.

Antimicrobial agent	Number of resistant bacteria	Resistance rate (%)
Piperacillin	29	65.91
Ampicillin/sulbactam	12	27.27
Ceftazidime	5	11.36
Cefpirome	6	13.64
Cefuroxime	14	31.82
Cefazolin	18	40.91
Cefotaxime	2	4.55
Ceftriaxone	14	31.82
Ertapenem	0	0
Cefotetan	1	2.27
Imipenem	0	0
Meropenem	0	0
Gentamicin	7	15.91
Levofloxacin	15	34.09
Ciprofloxacin	15	34.09

### 3.3 Comparison of baseline information between the training set and validation sets

The training set comprised 364 cases, with 55 cases of UTI; the validation set comprised 157 cases, with 27 cases of UTI. There was no statistically significant difference in baseline features between these two sets (*P* > 0.05; **Table 4**).

**Table 4:** Baseline characteristics in the training and validation sets.

Baseline information		Training set (N = 364)	Validation set (N = 157)	Statistical values	*P*-value
Sex [n (%)]	Male	167 (45.88)	59 (37.58)	3.076	0.079
	Female	197 (54.12)	98 (62.42)		
Age (years)		69.00 (64.00,76.00)	69.00 (63.00,75.00)	-0.622	0.534
BMI (Kg/m^2^)		25.20 (23.30,27.60)	25.20 (22.50,27.54)	-1.049	0.294
FBG (mmol/L)		9.00 (7.50,10.38)	8.80 (7.05,10.03)	-1.550	0.121
2h-PG (mmol/L)		14.50 (12.23,17.78)	14.97 (12.10,18.30)	-0.892	0.372
HbA_1_c (%)		9.20 (7.50,10.60)	9.30 (7.85,11.20)	-1.612	0.107
Scr (μmol/L)		60.05 (49.50,75.43)	59.20 (48.60,71.60)	-0.947	0.344
BUN (mmol/L)		6.70 (5.50,8.08)	6.60 (5.50,7.95)	-0.465	0.642
Serum albumin (g/L)		38.40 (35.70,40.30)	38.70 (36.15,40.30)	-0.823	0.410
Urine microalbumin (mg/L)		14.50 (5.23,55.35)	12.00 (4.90,43.00)	-0.507	0.612
Days of hospitalization (d)		8.00 (6.00,10.00)	7.00 (6.00,9.00)	-1.806	0.071
Duration of DM (year)		12.00 (6.00,17.00)	10.00 (7.00,15.00)	-1.434	0.152
SGLT-2i in previous year [N (%)]	Yes	103 (28.30)	53 (33.76)	1.560	0.212
	No	261 (71.70)	104 (66.24)		
Urine protein [N (%)]	Positive	73 (20.05)	33 (21.02)	0.063	0.802
	Negative	291 (79.95)	124 (78.98)		
Indwelling urinary catheter [N (%)]	Yes	17 (4.67)	10 (6.37)	0.644	0.422
	No	347 (95.33)	147 (93.63)		
UTI in previous year [N (%)]	Yes	33 (9.07)	18 (11.46)	0.715	0.398
	No	331 (90.93)	139 (88.54)		
Combined urinary stones [N (%)]	Yes	18 (4.95)	7 (4.46)	0.057	0.812
	No	346 (95.05)	150 (95.54)		

Continuous data are presented as the median (interquartile range, IQR) and compared using the Mann–Whitney U test.

### 3.4 Univariate logistic analysis of UTI in older patients with T2DM

The univariate analysis of the training set indicates that the infection group exhibited notably higher prevalence rates for the following categorical variables: female, SGLT-2i in previous year, indwelling urinary catheter, and UTI in previous year. For continuous variables, the infection group also demonstrated significantly higher HbA1c levels. (*P<* 0.05; **Table 5**).

**Table 5:** Univariate analysis of UTI in older patients with T2DM.

Variable		Non-UTI group (n = 309)	UTI group (n = 55)	Statistical values	*P*-value
Sex [n (%)]	Male	154 (49.84)	13 (26.64)	12.909	<0.001
	Female	155 (50.16)	42 (73.36)		
Age (years)		69.00 (63.00,76.00)	71.00 (65.00,76.00)	-1.296	0.195
BMI (Kg/m^2^)		25.10 (23.30,27.48)	26.81 (23.23,28.10)	-1.475	0.140
FBG (mmol/L)		8.90 (7.30,10.30)	9.33 (8.60,10.70)	-1.875	0.061
2h-PG (mmol/L)		14.50 (12.10,17.75)	15.00 (12.70,17.90)	-1.134	0.257
HbA_1_c (%)		8.90 (7.35,10.40)	10.50 (9.60,11.50)	-4.709	<0.001
Scr (μmol/L)		60.10 (49.50,74.90)	57.70 (48.20,76.80)	-0.027	0.978
BUN (mmol/L)		6.70 (5.50,8.00)	6.50 (5.50,8.90)	-0.226	0.821
Serum albumin (g/L)		38.50 (35.50,40.30)	38.00 (36.40,40.00)	-0.419	0.675
Urine microalbumin (mg/L)		13.70 (5.05,55.60)	17.10 (6.60,55.40)	-0.549	0.583
Days of hospitalization (d)		7.00 (6.00,10.00)	8.00 (6.00,11.00)	-0.957	0.338
Duration of DM (year)		12.00 (6.00,16.50)	15.00 (6.00,21.00)	-0.912	0.362
SGLT-2i in previous year [N (%)]	Yes	79 (25.57)	31 (56.36)	21.000	<0.001
	No	230 (74.43)	24 (43.64)		
Urine protein [N (%)]	Positive	60 (19.42)	13 (23.64)	0.518	0.472
	Negative	249 (80.58)	42 (76.36)		
Indwelling urinary catheter [N (%)]	Yes	10 (1.36)	7 (14)	9.446	0.002
	No	299 (96.76)	48 (86)		
UTI in previous year [N (%)]	Yes	19 (5.22)	14 (28)	21.108	<0.001
	No	290 (94.78)	41 (72)		
Combined urinary stones [N (%)]	Yes	16 (4.40)	2 (3.64)	0.236	0.627
	No	293 (95.60)	53 (96.36)		

### 3.5 Multivariate logistic analysis of UTI in older patients with T2DM

The multicollinearity analysis identified no correlation among the variables (VIF< 5; **Table 6**). The linear relationship of HbA1c was validated using a restricted cubic spline function (nonlinear test *P* = 0.981), and it was incorporated as a continuous linear variable into the predictors. On this basis, using the presence of UTI as the dependent variable, we conducted a multivariate logistic analysis with five factors statistically significant in univariate analysis (female, HbA_1_c, SGLT-2i in previous year, indwelling urinary catheter, and UTI in previous year) as independent variables. Per our findings, female (OR = 2.53, 95% CI: 1.25–5.12), HbA1c (OR = 1.35, 95% CI: 1.15–1.59), SGLT-2i in previous year (OR = 2.29, 95% CI: 1.19–4.41), indwelling urinary catheter (OR = 3.04, 95% CI: 1.02–9.23), and UTI in previous year (OR = 5.22, 95% CI: 2.22–12.25) were independent risk factors for UTI in older patients with T2DM (*P<* 0.05; **Table 7**).

**Table 6:** The result of collinearity analysis.

Variable	Female	HbA_1_c	SGLT-2i in previous year	Indwelling urinary catheter	UTI in previous year
VIF	1.025	1.021	1.008	1.030	1.020

**Table 7:** Multivariate analysis of UTI in older patients with T2DM.

Potential factors	β-value	SE	Wald value	*OR*	95%*CI*	*P-*value
Constant	−5.828	0.878	-6.635	——	——	——
Female	0.927	0.362	2.563	2.53	(1.25,5.12)	0.011
HbA_1_c	0.301	0.082	3.678	1.35	(1.15,1.59)	0.001
SGLT-2i in previous year	0.830	0.334	2.488	2.29	(1.19,4.41)	0.013
Indwelling urinary catheter	1.112	0.566	1.965	3.04	(1.02,9.23)	0.048
UTI in previous year	1.652	0.435	3.797	5.22	(2.22,12.25)	0.001

The final logistic regression equation was as follows: Logit (*P*) = −5.828 + 0.927 × female + 0.301 × HbA1c + 0.830 × SGLT-2i in previous year + 1.112 × indwelling urinary catheter + 1.652 × UTI in previous year.

### 3.6 Development of a prediction model for UTI in older patients with T2DM

**Figure 1:**
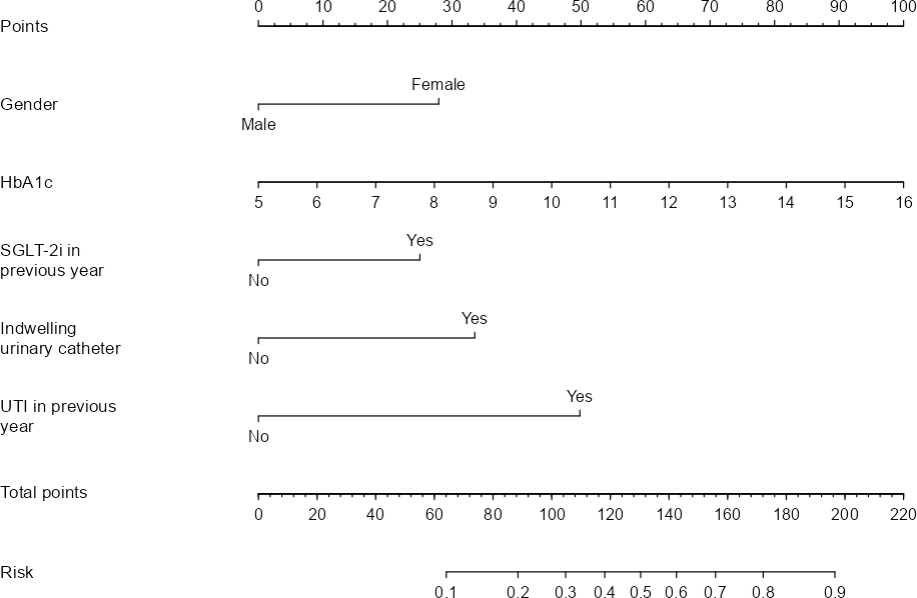
Prediction nomogram for older T2DM patients with UTI.

In this study, a nomogram model for predicting UTI in older patients with T2DM was established based on five independent risk factors: female, HbA_1_c, SGLT-2i in previous year, indwelling urinary catheter, and UTI in previous year. The corresponding scores of each independent risk factor were summed to obtain the total score, which corresponded to the probability of occurrence of infection in older T2DM patients with UTI (**Figure 1**). The nomogram assigns each variable a specific score on the top-point scale (range: 0–100), as illustrated in **Figure 1**. The cumulative score, derived from summing all individual variable scores, corresponds to the predicted probability of UTI indicated on the bottom risk scale. Notably, higher total scores are associated with an elevated risk of older T2DM patients with UTI.

### 3.7 Validation and evaluation of the prediction model for older T2DM patients with UTI

**Figure 2:**
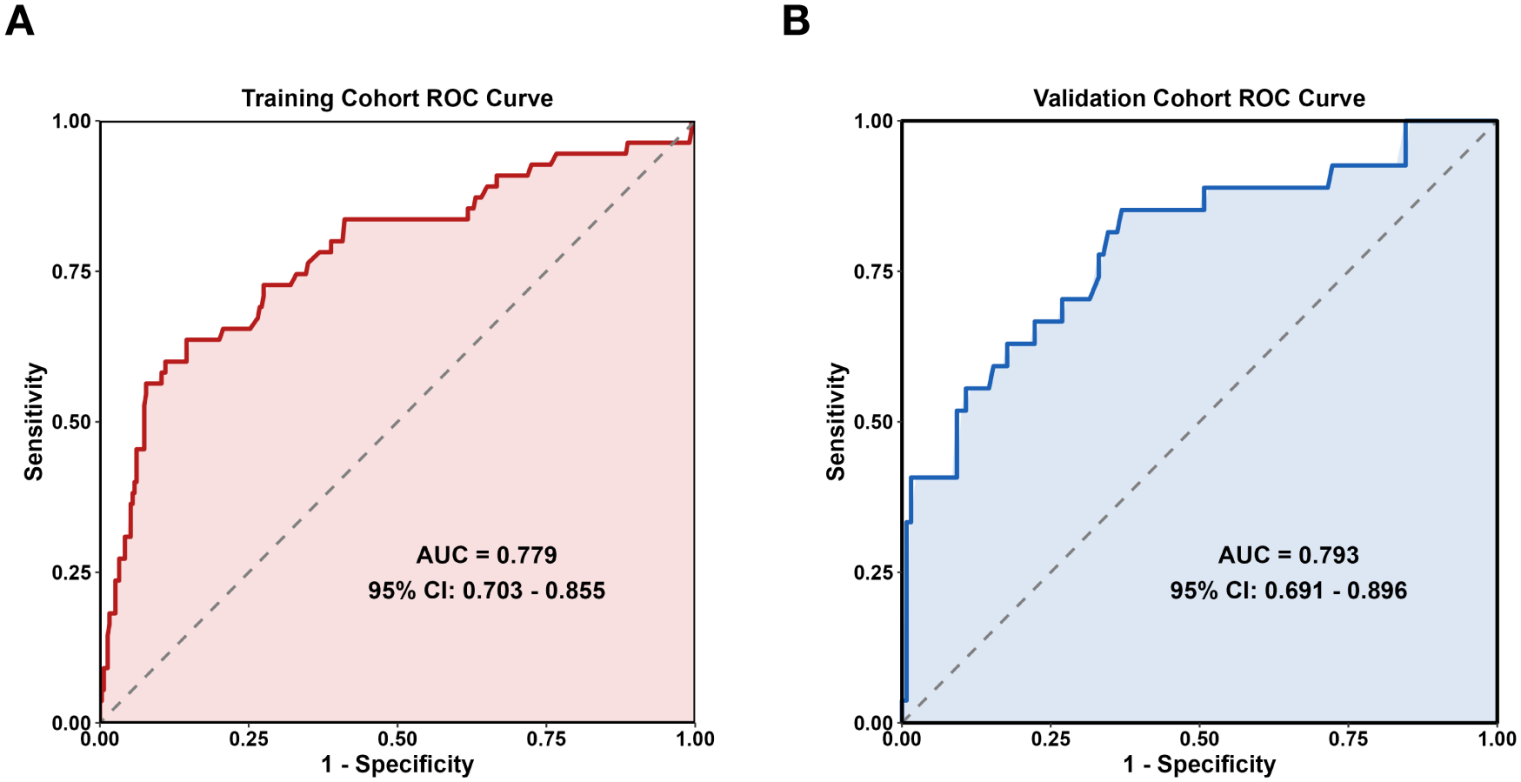
Prediction model ROC curve. **(A)** Training set **(B)** Validation set.

**Figure 3:**
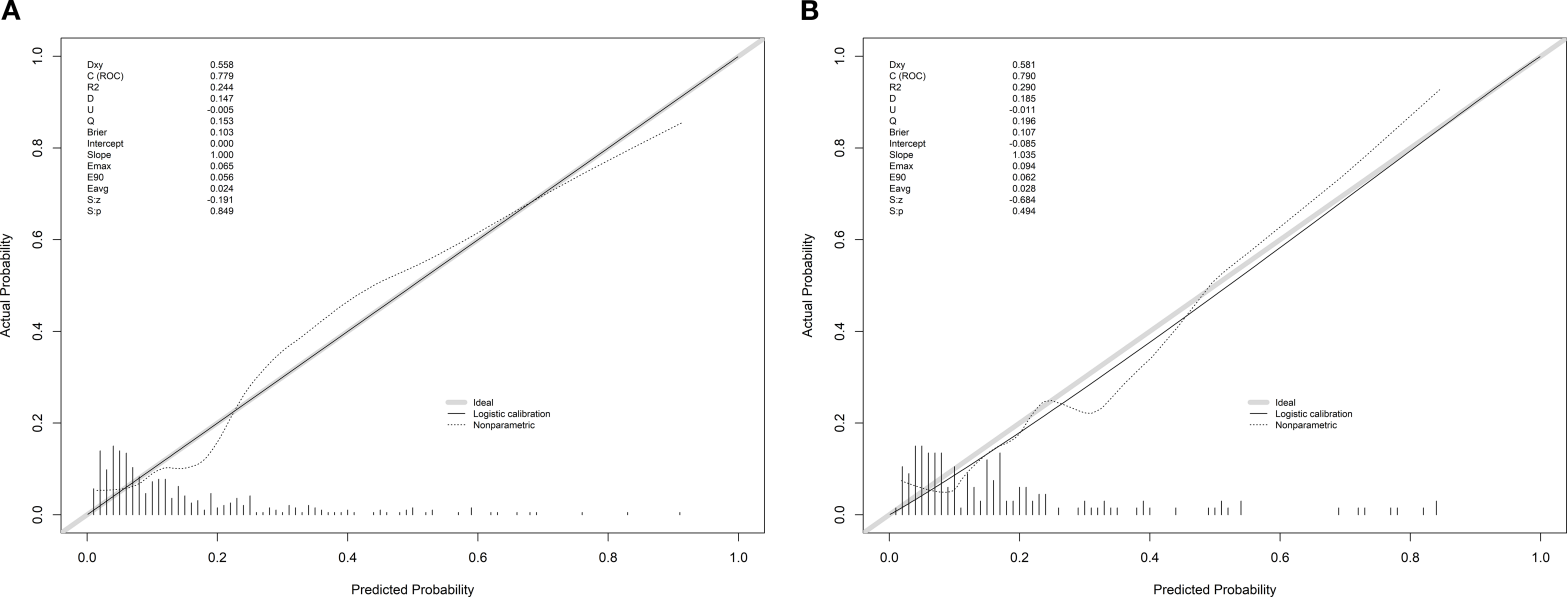
Calibration curve of the prediction model. **(A)** Training set **(B)** Validation set.

**Figure 4:**
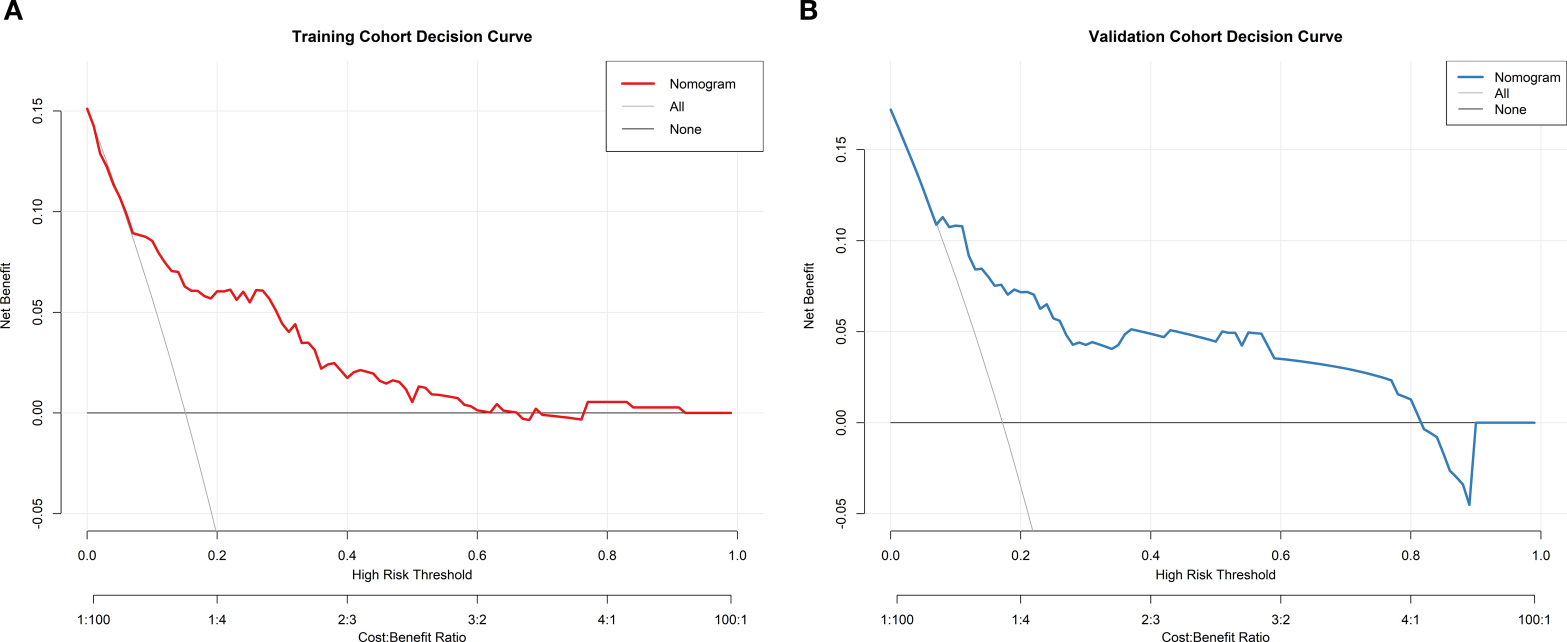
DCA curve of the prediction model. **(A)** Training set **(B)** Validation set.

The AUC of the training set was 0.779(95% CI: 0.703–0.855), and that of the validation set was 0.793 (95% CI: 0.691–0.896) (**Figures 2A, B**). As an AUC exceeding 0.75 indicates reliable discrimination, this nomogram demonstrated commendable performance in the training and validation sets. The H-L test results in the training (χ^2^ = 9.834, *P* = 0.277) and validation (χ^2^ = 5.432, *P* = 0.711) sets demonstrated good model fit. Internal validation of the nomogram prediction model’s training and validation sets was performed using the Bootstrap method (B = 1000). Calibration curve results indicated that the actual curves, calibrated curves, and ideal curves of the training and validation set models were well calibrated (**Figures 3A, B**). The model’s DCA curve suggested good clinical applicability of this risk prediction model (**Figures 4A, B**).

### 3.8 Example of nomogram application for individual risk estimation

Assume an older female patient with T2DM has the following parameters: HbA1c of 10%, used SGLT-2i in previous year, no indwelling urinary catheter, and UTI in previous year. According to the nomogram in **Figure 1**, the predicted probability of this patient developing a UTI is approximately 64.4%. Based on the DCA curve, this patient requires preventive intervention to mitigate her UTI risk.

## 4 Discussion

In this study, the pathogenic bacteria in older T2DM patients with UTI were predominantly Gram-negative bacilli (78.62%), with *Escherichia coli* accounting for 49.44%, which was in line with the findings of previous studies (24–26). The drug sensitivity results showed the high resistance of *Escherichia coli* to commonly used antibiotics such as piperacillin and cefazolin, but fully susceptible to carbapenems and other β-lactams. Most of the detected pathogenic bacteria were conditionally pathogenic, reflecting the immune, metabolic, and microenvironmental disorders in older T2DM patients with UTI (27); therefore, older T2DM patients with UTI need to use antimicrobial drugs rationally, according to the drug sensitivity results.

In recent years, owing to the aging of the population and the increasing incidence of T2DM (5), the incidence of its complications has been on the rise. Older patients with T2DM are highly predisposed to the risk of UTI, and, in this study, the incidence of UTI among older patients with T2DM was 15.74%; therefore, understanding the independent risk factors for UTI in older patients with T2DM is essential for UTI prevention and the improvement of patients’ quality of life. Relevant studies have demonstrated that there are more independent risk factors for UTI among older patients with T2DM (24–26, 28). Based on the risk factors reported in previous studies, we mobilized the clinical data of older T2DM patients, identified the independent risk factors for UTI in older patients with T2DM more comprehensively, and developed a nomogram prediction model.

The susceptibility of older female patients with T2DM to UTI may be associated with the unique structure of the female urinary system (29). Additionally, postmenopausal older female patients with T2DM have decreased estrogen levels, leading to urethral mucosal atrophy and a shift in vaginal pH toward alkalinity. This disrupts the normal vaginal flora, increasing the risk of UTI (30). HbA1c reflects overall glycemic control over the preceding 2–3 months (31), and prolonged hyperglycemia, which manifests as high HbA1c, impairs immune function (32). Elevated plasma osmolarity during hyperglycemia reduces antimicrobial protein concentrations and enhances bacterial proliferation (33). Hyperglycemia causes microvascular lesions in the autonomic nerves supplying the urinary tract, leading to autonomic neuropathy. This damages neural pathways and contributes to neurogenic bladder pathogenesis (34). In older patients with T2DM, blood glucose levels exceeding the renal threshold result in “high sugar” urine, collectively increasing the likelihood of UTI in this population. Older T2DM patients with indwelling urinary catheters are prone to risk of UTI. Catheter placement may damage the urethra and disrupt the normal physiological barrier, leading to infection. In contrast, the catheter creates a direct connection between the urethra and the external environment, increasing exposure to pathogens. This is particularly true for patients with long-term catheterization, where prolonged catheter placement irritates the urethral mucosa, causing edema and exudation, predisposing the urethra to pathogen invasion (35). T2DM patients with UTI are prone to recurrence and are hard to treat. Pathogenic bacteria may persist in the urinary tract as opportunistic pathogens, leading to recurrent infections when patients with T2DM experience impaired immune function or dysbiosis (36).

In this study, SGLT-2i in previous year was identified as a risk factor for UTI in older patients with T2DM. This association may be related to SGLT-2i reducing renal glucose reabsorption, thereby increasing urinary glucose excretion and creating a favorable environment for bacterial growth and proliferation (37). However, the role of SGLT-2i as a risk factor for UTI remains controversial (38). The U.S. Food and Drug Administration (FDA) has issued a drug safety communication regarding an increased risk of severe UTI associated with SGLT-2i use (38). Furthermore, a 2013 systematic review found that SGLT-2i use was associated with a 34–42% increased risk of UTI (39). However, ongoing research and systematic reviews have since concluded that SGLT-2i use does not notably increase the incidence of UTI in patients with T2DM (22, 40), which contradicts the findings of this study. The reasons for this discrepancy are analyzed as follows: (1) Previous systematic reviews included T2DM patients aged ≥18 years, with a high proportion of middle-aged and young adults who generally have fewer baseline comorbidities and stronger immune function; This study, however, focused on older patients with T2DM, who often have a longer disease duration, higher risks of diabetic nephropathy and cardiovascular disease, and frequently experience physiological changes such as reduced immune function and impaired bladder function, resulting in a higher baseline UTI risk. Additionally, only five classes of SGLT-2i are approved in China (canagliflozin, dapagliflozin, empagliflozin, ertugliflozin, and henagliflozin). Clinicians guided by expert consensus prefer dapagliflozin, empagliflozin, and canagliflozin to enhance cardiovascular and renal protective effects in older patients (41). However, multiple subgroup analyses have confirmed that certain SGLT-2is (e.g., dapagliflozin and empagliflozin) markedly increase UTI incidence (22, 40, 42). (2) Previous studies have primarily been randomized controlled trials (RCTs) in which patients received standardized follow-up monitoring during the study period. In contrast, this retrospective observational study accurately reflects the clinical practice setting for older patients with T2DM. Poor medication adherence in this population may further increase the risk of UTI. Additionally, older patients with T2DM typically have a longer duration of diabetes and higher number of comorbidities, which are, themselves, strong risk factors for UTI. RCTs balance these factors through randomization.

The model demonstrates moderate discriminatory ability on the training and validation sets, though its AUC values fall below those reported for machine learning methods by Yu Xiong et al. (17). However, unlike complex machine learning models, this model integrates only five readily accessible clinical variables, making it more suitable for rapid assessment in routine clinical settings. The model’s discriminatory power is comparable to that of the questionnaire-based model by Venmans et al. (14, 15), but it offers a more comprehensive evaluation. In contrast, while the model by Tu Jing et al. (16) demonstrates excellent discriminatory ability, it suffers from severe sample size inadequacy and lacks assessments of calibration and clinical utility. This study rigorously adheres to sample size requirements and is subjected to comprehensive validation and internal validation, thereby enhancing methodological rigor and reliability. According to this nomogram model, clinicians should prioritize screening high-risk patients and monitoring their infection status. Clinicians should clearly define indications for catheter use and its critical importance. Nursing staff must strictly adhere to indwelling catheter care protocols, prioritize early catheter removal, and rigorously enforce hand hygiene management. When UTI occurs, empirical antimicrobial therapy can be initiated promptly based on the antimicrobial susceptibility results of *Escherichia coli* from this study, while awaiting urine culture results to adjust the treatment regimen accordingly (43). The Chinese Diabetes Association recommends HbA1c control<7% for most adult patients with T2DM (44). Given the complex individual conditions and frequent organ dysfunction in older patients with T2DM, personalized adjustment of glycemic control targets is necessary (18). Patients should prioritize self-management of blood glucose levels, strictly adhere to prescribed hypoglycemic medications, control their diet, and closely monitor blood glucose levels.

Nevertheless, this study has several limitations: (1) This single-center observational study carries the risk of unmeasured confounding. Although common risk factors were adjusted for in the multivariate analysis, unrecorded variables may have influenced the results. (2) Clinical data may contain a potential recall bias, particularly for data on “UTI in previous year,” which relied on retrospective medical record review. (3) The definition of “SGLT-2i in previous year” is ambiguous and does not differentiate between different formulations and doses of the medication. (4) The outcome variable is UTI, without distinguishing specific subtypes such as asymptomatic bacteriuria or cystitis. (5) Although the sample size meets the minimum requirement for prediction model establishment, EPV is approximately 11, which is on the lower boundary of the acceptable range. This may affect the stability of model coefficient estimation to a certain extent. (6) The model lacks external validation, limiting its general applicability to other healthcare institutions or regions. In view of these limitations, future research should prioritize prospective validation in multicenter cohorts encompassing hospitals across diverse regions. Additionally, these studies should focus on refining variable definitions, conducting stratified analyses of UTI by clinical subtypes, and further exploring pathogen-specific risk patterns.

## 5 Conclusions

In summary, we developed a simplified prediction chart to assess older T2DM patients with UTI based on five readily accessible clinical variables, including the female, HbA1c, SGLT-2i in previous year, indwelling urinary catheter, and UTI in previous year. Internal validation demonstrated the model’s strong discriminatory performance. Furthermore, *Escherichia coli* was identified as the predominant causative pathogen, exhibiting a distinct pattern of antimicrobial susceptibility. Integrating this predictive tool with local pathogen susceptibility data could form a practical clinical strategy for early risk stratification, guiding preventive measures and supporting targeted empirical therapy. External validation through multicenter cohorts is required before widespread clinical implementation.

## Data Availability

The raw data supporting the conclusions of this article will be made available by the authors, without undue reservation.
