# Correction: Development and validation of a prediction model for urinary tract infection in older patients with type 2 diabetes mellitus

**DOI:** 10.3389/fendo.2026.1863109

**Published:** 2026-05-06

**Authors:** Lin Yang, Jian Yin, Zhengxiong Yang, Jing Liu

**Affiliations:** 1 Hospital-Acquired Infection Control Department, The Affiliated Lianyungang Hospital of Xuzhou Medical University, Lianyungang, China; 2 Department of Endocrinology, The Affiliated Lianyungang Hospital of Xuzhou Medical University, Lianyungang, China

**Keywords:** nomogram, older, prediction model, risk factors, type 2 diabetes mellitus, urinary tract infection

There was a mistake in **Figure 2** as published. The original ROC curve of Figure was plotted using a smoothed method, which resulted in slight discrepancies in the reported AUC values and 95%CI. The ROC curve has now been recalculated and replotted using the correct unsmoothed method, resulting in a slight increase of 0.015 in the AUC on the training set and a slight increase of 0.014 on the validation set, and the 95% confidence intervals have been updated for the training and validation cohorts. The corrected **Figure 2** appears below.

**Figure 2:**
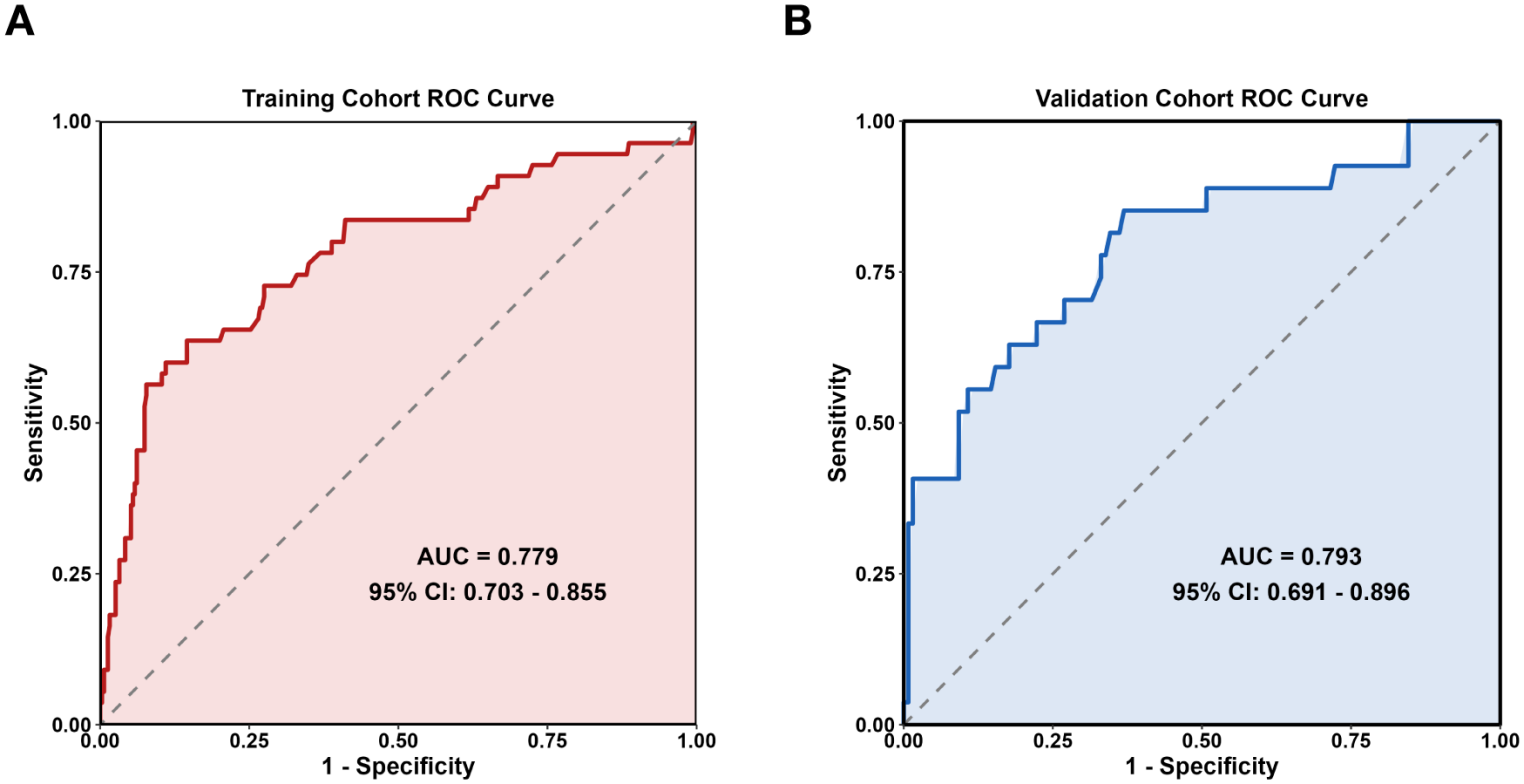
Prediction model ROC curve. **(A)** Training set **(B)** Validation set.

There was an error in the **Abstract**. “The AUC was 0.764 (95% *CI*: 0.683–0.898) in the training group and 0.779 (95% *CI*: 0.703–0.855) in the validation group.” has been corrected to read:

“The AUC was 0.779 (95% *CI*: 0.703–0.855) in the training group and 0.793 (95% *CI*: 0.691–0.896) in the validation group.”

In the published article, there was an error in the **Results** section, sub-section 3.7. “The original ROC curve analysis in **Figure 2** was generated using a smoothed plotting method, which led to minor deviations in the reported AUC values and 95%*CI*.” has been corrected to read:

“The AUC of the training set was 0.779(95% *CI*: 0.703–0.855), and that of the validation set was 0.793 (95% *CI*: 0.691–0.896) (**Figures 2A, B**).”

The original version of this article has been updated.

